# Supplementary material for: Patterns of Intron Gain and Loss in Fungi
Source: PLoS Biol. 2004 Nov 30;2(12):e422. doi: 10.1371/journal.pbio.0020422 (PMC532390; doi:10.1371/journal.pbio.0020422)
Supplement: Table S1 — Also available at http://genes.mit.edu/NielsenEtAl/. (4.3 MB ZIP). [file pbio.0020422.st001.zip › NielsenEtAl/html/23.html]

AN7576.1.NCU02689.1.MG04377.1.FG01204.1


```
 CLUSTAL W (1.82) Multiple Sequence Alignments - Introns Inserted


Sequence 1: NCU02689.1	1279 aa
Sequence 2: MG04377.1	1181 aa
Sequence 3: FG01204.1	1077 aa
Sequence 4: AN7576.1	1201 aa
Alignment Length: 1347 aa
Number Identitical Residues: 428 aa
Alignment Score (without introns) 22829


MG04377.1 	---------------------MSAPDPFDGPPADRGRGDRLALGE-ENYQMDRENALQLS
NCU02689.1	---------------------MASSDYGGGPVGDREYTHGHPQGHPQGEVAHRREDEHLD
FG01204.1 	----------------------MSSSYDDAMLGEHSHG--------EHSHQYHAQDMPPQ
AN7576.1  	MPPGDVSLPDSLVPGNGAVRPSLNTSGYGGSNGGSFQTQTPTSPADSTVPFDSPRTRPWN
          	 .... : ..:  ....:  .   ..  ..  .        .    .       .    .

MG04377.1 	MGAPRQNGG-AYPSAVPPGAPSPAWSAGGDRLRDERSR-----SAERNRSRPRN---GRS
NCU02689.1	ITPTISQNG-APTNAHLTRIPGSPALSAADATDLESRR-----SGEKNRSKPKG---SRT
FG01204.1 	ADSNWQQNG-NFR----TRTPDS-------------------------SYRPRP---PIS
AN7576.1  	ASSTGPDSGQSPVDGRPIRRLDSNPVDARDPTTPQDRTNHWENSGPLERPRPNGRPHAKS
          	  .   :.*.   ..      ..    . .    .   .  ..:.  .  :*.  .   :

MG04377.1 	ASGQLRICAKCGEPLTGQFVRALDGTFHLDCFKCR0DCGQIV~ASKFFPAEDENGGGQYP
NCU02689.1	ASGQVRVCKKCGEPLTGQFVRALDGTFHLDCFKCR0DCGQIV~ASKFFPADDNNGEGQYP
FG01204.1 	AP---------------------------------~-----V1ASKFFPAEDENGQGQYP
AN7576.1  	PGSSSRICKKCNEPLTGQFVRALMATYHLECFKCE0DCGQIV~ASKFFPVDAEDGSGQYP
          	. ..   . ..... :..   :  .:   .. ..  .... * ******.: ::* ****

MG04377.1 	LCEKDYFRRLGLLCYQCGNALRGSYITALDRKYHVDHFTCSLCDTVFGAQDSYYEHDGSV
NCU02689.1	LCETDYFRRLGLLCYQCGGALRGSYITALDRKYHVDHFTCSLCPTVFGAQDSYYEHDGHV
FG01204.1 	LCETDYFRRLGLLCYQCGGALRGSYITALDRKYHVDHFTCSLCSTIFGAQDSYYEHDGNV
AN7576.1  	LCEIDYFRRLDLLCHDCGGALRGSYITALDRKYHIEHFTCSVCPTVFGAQDSYYEHEGKV
          	*** ******.***::**.***************::*****:* *:**********:* *

MG04377.1 	YCHYHYSTQFAQKCNGCQTAILKQFVEIYRNGQNQHWHPECYMIHKFWNVRLTPPQEASA
NCU02689.1	YCHYHYSTQYAQRCSGCQTAILKQFVEIFRNGQNQHWHPECYMIHKFWNVRLNPPQ--KA
FG01204.1 	YCHYHYSTQFAQRCNGCQTAILKQFVEIFRNGQNQHWHPECYMIHKFWNVRLAQPADSVP
AN7576.1  	YCHYHYSTQFAQRCHGCHTAILKQFVEIFRNGQNQHWHPECYMIHKFWNVRLAPTGQPLE
          	*********:**:* **:**********:***********************  . ..  

MG04377.1 	LSLDQDEQAS--RELVKVGEERMEEKVYRIWSTLSTFEESSAACISDMLLHVSNGAYVDG
NCU02689.1	LGPVPDDPAG--RELVRDEEERMEEKVYRIWSVLSTFEESSAACISDMLLHVSNGAYVDG
FG01204.1 	ALESTDDAAG--RDLIREEEERMEEKVFRIWSVLSTFEESSAACISDMLLHVSNGSYVEG
AN7576.1  	YPQMKEDASDEERNQIREEEDLMEEKVYKIWSTLSTFEESSAACISDMLLHVSNGAYLDG
          	     :: :...*: ::  *: *****::***.**********************:*::*

MG04377.1 	VMVAKKFIWHVDILFKSADGLDAAMTKLEMKA1LSYGREAKLLCKKIVAFFSLLSKAQDK
NCU02689.1	VMVAKKFIWHVDILFHSADRLDATMERLDMTG1LMYGREAKLLCKKIVAFFSLLSKTQDK
FG01204.1 	VFVAKRFIYHVEILFQSADRLDATMVGLDQKG1MSYGREAKLLCKKIVSFFSLLSKTQDN
AN7576.1  	VLVAKRFIGHVEILFSAVDELAVSIKAQGMKD1LAFGREAKLLCKKIVAFFALLSKTQET
          	*:***:** **:*** :.* * .::     .  : :************:**:****:*:.

MG04377.1 	EVRKLGVTQELLSLVTGLAHYLKLLIRICLQGALRIEKERNTSDGLYQFLDDLGNLESLK
NCU02689.1	GARKLGVTQELLTLVTGLAHYLKLLIRICLQGALRIEKERDTADGLDGFLDELNDLEALK
FG01204.1 	ATHKIGVTQELLSLVTGLAHYLKLLIRICLQGALRLEREAHSSTGLYQFLDDLSDLDATK
AN7576.1  	GVKKLGVTQELLSLVTGLAHYLKLLIRIGLQGALKLEREQQSPEGLHSLLDRLGDLESLR
          	 .:*:*******:*************** *****::*:* .:. **  :** *.:*:: :

MG04377.1 	GEEQS---LSITMGMSRLSANDSDQCSLCRKPIEDECAKNEEKRWHIA--CVACNRCGRE
NCU02689.1	TEDNSTTTLQLTSGMARLSASDSDSCTLCKKPIEDECAKSGDKRWHIT--CVNCSRCQKE
FG01204.1 	NDDGQ---LSVTASGGKLSANDSDHCALCHRSVEDECAKNADMRWHLN--CVRCTKCSRD
AN7576.1  	PLEEEETPADLLVGVDTLADQLSDCCAACKEPIDDECVMLGDNRWHIKPPHFLCSSCQTD
          	  : . :. .:  .   *: . ** *: *:..::***.   : ***: .. . *. *  :

MG04377.1 	LGRSLHDAQFNPYDQKIFCTNCGSFY---ADNAPPFTRVTKLQQYVYLLKVALARLLEIL
NCU02689.1	LGRSLQDARLNLYDSKIFCNNCETYS---SDKAPPFERVSKLQQYMFLLQVALARLLEIL
FG01204.1 	IGRSLAEARLNTLDRKLYCTNCAGP------NAQSLEHVSKLQQYVFLLRVALARLLDIL
AN7576.1  	LTTNLQDAFLSPKDKQILCSSCVTHKGIVNNTQKGFIRVTKLQQFVFLLRVALARLLAVL
          	:  .* :* :.  * :: *..*    .   ..   : :*:****:::**:******* :*

MG04377.1 	RSTGAIPRED~GHPQD--LHDGQRDLPP-HLRSEARSKSFAAG--SMHQRESSYESAVND
NCU02689.1	RMHGALP--S~AAEAR--GHNGIDAGDG-RPLMDGRQRGFPGGDQSLHHRESSYESALND
FG01204.1 	KANGTLPRSN~DDSDN--NGFGASDGPGGDPKSRGGDQSRGPG---GSKRESSYENTLND
AN7576.1  	RAGGTLFPPE1TDMPSSFVEDSVVQSDGNQIPPGGELRRRKDG------DGSSLEQTVGE
          	:  *::   .      :    .      .     .  :    *        ** *.::.:

MG04377.1 	VKRLKSTRLDKALSSSVRKARTSRIMEGPDGTAGRPGS---AGANSGDPRKPGFSIVE--
NCU02689.1	VKRLKSTRLDKHLSSSFRKARTSRIMDGPESSSAQPGSGPGSGIDGGPANGRRMRIEE--
FG01204.1 	VRRLRSTRLDRHLSSSVRQARSSRVMD-TDGRGPRPGS-------AGDASQ---------
AN7576.1  	MRRLRSIRNERTLSTTYKKARASRIIDGPEGRSVRPGS---SGGEGSDSRGPGFQIVEEK
          	::**:* * :: **:: ::**:**:::..:. . :***   :. ... .        ...

MG04377.1 	DPANERAEDSMLGNQDALTLDDIPRIVAAEQAREQRPNAFQRQELFRAPATEPNLN----
NCU02689.1	DMGPGDDHDMMLPNQDALTLADIPRIVAAEQAREQRTGPPSRHELLRSPATEPSFGGASS
FG01204.1 	--GGQNSTDLMFGQNDGITLDDIPRIVAVEQSREQQQRVPQP---FQDRSLDAPSG----
AN7576.1  	DANGEPVTELAFGNQDALTLDDIPRIVAAEQAKEQRPNAYRHAGTKLVGTTEPLPRYN--
          	.       :  : ::*.:** *******.**::**:             : :.       

MG04377.1 	-SNNPRAFSPGRG--PRSHGEPSPQRGMVMGIGGRKYFSELSNLDYFIVRHLAVLTMHPL
NCU02689.1	GSGHKRSYSDSKGGDPRAAGEPSPQR------VGRRYFSELSALEYFIIRHLAVVTMAPM
FG01204.1 	-QGHQRTQSNDRE---RSD---MPRQ------IGRKFFSELSGLEYFNVRHLAVLTMQPM
AN7576.1  	-QGHQRGVS-NAG---IEQPYPAPTG------RGKRYFSELSALEYFIVRHVAVLSMEPL
          	 ..: *  * .          . *         *:::***** *:** :**:**::* *:

MG04377.1 	LNNEFELEELLGFIESRKPATFWNKFGKAFKNDGKKNVKKKGVFGVPLEMIIERDGADST
NCU02689.1	VEGEFTLVELIGLIETRKTPNFWKNIGKAFQKDGRK---KKGVFGVPLEAIIERDGAEST
FG01204.1 	IEQEFTLDELLSFIESRKQATFWKNIGKAFKNDKNKSVKKKGVFGVPLEIIIERDGADST
AN7576.1  	LEGAFTLEELLSLIESRK-PTIWGIFGRAFTKDAKKGGKKKGVFGVSLDYLVEKEGTEST
          	::  * * **:.:**:** ..:*  :*:** :* .*. .*******.*: ::*::*::**

MG04377.1 	DGVGPGTLRIPAIVDDIVATMRQMDLSVEGVFRKNGNIKKLSELCEKIDREGCDSVNLSS
NCU02689.1	DGVGPGTLRIPAVIDDILSLMRQMDLSVEGVFRKNGNIKKLGELVEKIDKEG--NVDLSG
FG01204.1 	DGVGPGTLRIPAVVDDIVTSMRKMDLSVEGVFRKNGNIKKLQGLVETINTEGCDMVSFME
AN7576.1  	HGVGPGALRIPALVDDSVSAMRQMDMSVEGVFRKNGNIRRLKDISEMIDTKY-EQVDLTK
          	.*****:*****::** :: **:**:************::*  : * *: :  . *.:  

MG04377.1 	QPVVQVAALLKRYLRDLPDPLMTHKLYNLWLTAAK~IQDADKRRQCLHLICCLLPKCHRD
NCU02689.1	NNVVQVAALLKRYLRELPDPLMTQKLYRLWLAAAK~ISDEDKKRQCLHLICCLMPKCHRD
FG01204.1 	QPVVQLAALLKRYLRDLPDPLMTHKLYRLWISAAK~ISDHEKRKQCLHLTCCLLPKSHRD
AN7576.1  	ENPVQIAALLKKFLREMPDPLLTFKLHRLFVASQK1IPDLEKQKRVLHLTCCLLPKAHRD
          	:  **:*****::**::****:* **:.*:::: * * * :*::: *** ***:**.***

MG04377.1 	CLEILFCFLKWAGTFHQVDDESGSKMDVKNLATVIAPNILYLQSKALTLDSDPMFAIVAV
NCU02689.1	SMEILFCFLKWAGSFHQVDEESGSKMDIKNLATVIAPNVLFDRTATSTLGSDPMYAIEAV
FG01204.1 	CLEILFSFLKWAGTFHQLDEDLGSKMDVRNLATVIAPNILTNPTKAMAMDSEAMYIIDAV
AN7576.1  	TMEVLFAFLNWTSSFSHVDEETGSKMDIHNLATVMTPNILYPNTKTSAVD-ESFLAIEAV
          	 :*:**.**:*:.:* ::*:: *****::*****::**:*   : : ::. :.:  * **

MG04377.1 	ETLIQDIEEMCL0VPDDLVDILSDSTLFNNNGDLTTKEILKRFGDRPLMNGGLRG--HGD
NCU02689.1	EVLIANIEEMCL0VPDELVQYISDPAFLD--GELTTKEILKRYSERNTGGFGSRQ--YAD
FG01204.1 	EMMITNIEEMCQ0VPDEILGLMNDPYIFSNSGDLTTKDILKRFQDR----RGQIS--IAD
AN7576.1  	NALITYNDTMCE0IPEDLQSVLSDSHLFKENSEVTTKDILKRYGDIARGGFSQKASNGGE
          	: :*   : **  :*:::   :.*. ::....::***:****: :    . .   :. .:

MG04377.1 	--ELVGRHDTPNRPFARRVDTDP---AVWQDERSVRPVHDQQQYASS-------------
NCU02689.1	VVEIVGRHENPSRPPPRRVESDP---TSWQQETSVRTVTENAAYLGPGPTSGGGGGGGGG
FG01204.1 	VNEVYNRQDNSTRPPARRVETDP---AVWSGEATVRTIQDPSMPLPNN------------
AN7576.1  	TFTITNQSRGANVPTSARIETDPYQDTASQMQSSVRHVPGPG------------------
          	   : .:   .. * . *:::** ..:  . : :** :                      

MG04377.1 	--------------------------------MHNTPLRRHEDNG--------QPSPYG-
NCU02689.1	GSGGNNGGGMVDRTGGPGGAVSPQKRGGPGGGMGPHPLSQSHGDGGGPGGPQQQPIPYGP
FG01204.1 	--------------------------------GNATPPARFRGRN------DLNPSPYGQ
AN7576.1  	--------------------------------GNSHPSGAIAPNK---------------
          	                                    *                       

MG04377.1 	-----------------------------------------QDFDPSPGMSRE------P
NCU02689.1	APEHVAAAALAGSGGGGPGVGGQGGNAVGVGGGDGAPGGGGGDYNRNGGSTTSGLHNHNG
FG01204.1 	-----------------------------------------NQFNQSDSQLDS-------
AN7576.1  	------------------------------------------DLDKRTRSTSTG------
          	                                          : :        .      

MG04377.1 	SSDRGGGQRREWRNSGWGNNSRQPGSVGVTGTG
NCU02689.1	QQQQGGGPRKEWRNSGWG----RPNNGVTTGTA
FG01204.1 	---AEHSRRREWRNSGIG--GRQGSGLGVSGNS
AN7576.1  	--SQSEGQPQQVPYRARP----NPGPMGVAG--
          	  .   .  ::    .      . .   .:*
```
